# Short-Term and Long-Term Blood Pressure Changes and the Risk of All-Cause and Cardiovascular Mortality

**DOI:** 10.1155/2019/5274097

**Published:** 2019-08-06

**Authors:** Yue Dai, Yali Wang, Yanxia Xie, Jia Zheng, Rongrong Guo, Zhaoqing Sun, Liying Xing, Xingang Zhang, Yingxian Sun, Liqiang Zheng

**Affiliations:** ^1^Department of Clinical Epidemiology, Library, Shengjing Hospital of China Medical University, Shenyang 110004, China; ^2^Department of Cardiology, Shengjing Hospital of China Medical University, Shenyang 110004, China; ^3^Institute of Chronic Disease, Liaoning Provincial Center for Disease Control and Prevention, Shenyang 110005, China; ^4^Department of Cardiology, The First Affiliated Hospital of China Medical University, Shenyang 110001, China

## Abstract

**Background:**

Few studies compared the effects of BP changes in short- and long-terms on all-cause mortality and CVD mortality.

**Methods:**

We performed a 12.5-year follow-up study to examine the association between short- (2008 to 2010) and long-term [baseline (2004-2006) to 2010] BP changes and the risk of mortality (2010 to 2017) in the Fuxin prospective cohort study. The Cox proportional hazards model was used for this study, and the average BP was stratified according to the Seven Joint National Committee (JNC7).

**Results:**

We identified 1496 (805 CVD deaths) and 2138 deaths (1222 CVD deaths) in short- and long-term study. Compared with BP maintainer, in short-term BP changes, for participants from normotension or prehypertension to hypertension, the hazards ratios (HRs) and 95% confidence intervals (CIs) of all-cause mortality were 1.948 (1.118-3.392) and 1.439 (1.218-1.700), respectively, while for participants from hypertension to prehypertension, the HRs (95% CIs) were 0.766 (0.638-0.899) for all-cause mortality and 0.729 (0.585-0.908) for CVD mortality, respectively. In long-term BP changes, for participants from normotension or prehypertension to hypertension, the HRs (95% CIs) of all-cause mortality were 1.738 (1.099-2.749) and 1.203 (1.023-1.414), and they were 2.351 (1.049-5.269) and 1.323 (1.047-1.672) for CVD mortality, respectively. In addition, the effects of short-term BP changes on all-cause and CVD mortality, measured as regression coefficients (*β*), were significantly greater than those in long-term change (all* P*<0.05).

**Conclusions:**

Our study emphasizes that short-term changes in BP have a greater impact on all-cause and CVD mortality than long-term changes and assess the cut-off value of the changes in blood pressure elevation.

## 1. Introduction

In 2017, the global adult mortality rate has declined slowly; not only that, but in some cases, the mortality rate is still rising [1]. Noncommunicable diseases accounted for 73% of the total number of deaths worldwide, more than half of which were attributed to only four risk factors, and hypertension was one of them [2]. The relationship between blood pressure (BP) level and mortality (all-cause or cardiovascular disease (CVD)) has been investigated in numerous studies [3–7]. It is well known that BP is an ever-changing variable in individuals during follow-up [8]. Therefore, office BP at a single point in time did not accurately predict all-cause mortality and researchers have begun to pay attention to the relationship between BP changes and death risk in recent years. For example, Fan JH et al. [9] found that, relative to stable BP of normotension, having a rise in BP from normotension or prehypertension to hypertension both conferred an increased risk of total and CVD and stroke mortality. Susanne M et al. [10] identified 4 BP trajectories and found that ten-year BP trajectories were the strongest predictors, among different BP measures, of CVD and all-cause mortality. In Kim MK's study [11], they found that the risk of cardiovascular outcomes was increased with greater variability in systolic blood pressure (SBP) and greater BP variability leads to greater cardiac and vascular damage [12]. However, these studies focus on the impact of BP on outcomes at the same period, without considering both short- and long-term BP changes in one study. Short- and long-term BP changes may have different effects on mortality and it is unclear whether short- and long-term changes in BP categories are differentially associated with mortality risk.

In our study, we focused more on the changes in BP, a particularly compelling and underreported putative all-cause mortality risk factor, and to evaluate the relationship between changes in BP categories and all-cause and CVD mortality in a representative natural population.

## 2. Methods

### 2.1. Study Population and Study Design

**Figure 1:**
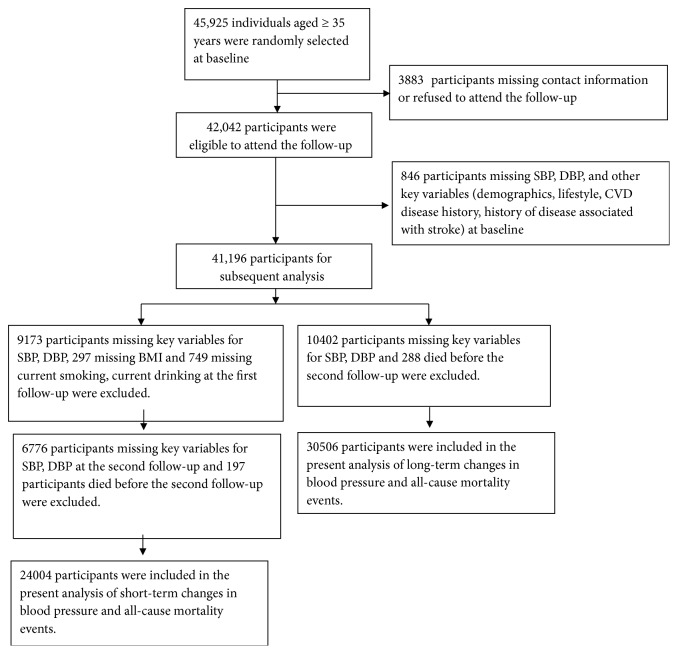
The study population inclusion and exclusion process.

This is a large-scale epidemiological follow-up study. From 2004 to 2006, a multistage, random cluster sampling design was performed to select a representative sample of the rural population aged 35 years and older from Fuxin County of Liaoning Province. The detailed methodology was described elsewhere [13]. From January to July 2008 (follow-up 1), from July to December 2010 (follow-up 2), and from March to December 2017 (follow-up 3), investigators were invited to participate in the follow-up study. Of the 45,925 participants at baseline, 3,883 subjects missed contact information or refused to attend the follow-up, and 42,042 participants were eligible to attend the follow-up. Of these, 846 participants who were missing SBP, diastolic blood pressure (DBP), or other key variables (demographics, lifestyle, CVD disease history, and history of disease associated with stroke) at baseline were excluded. For short-term changes analyses, subjects with missing SBP, DBP, and other key variables and missing body mass index (BMI), current smoking, and current drinking at the follow-up 1 (n=10,219) and missing SBP and DBP at the follow-up 2 (n=6776) and who died before the follow-up 2 (n=197) were excluded, leaving 24,004 participants for analysis. For long-term changes, subjects with missing SBP, DBP, and other key variables at the follow-up 2 (n=10,402) and who died before the follow-up 2 (n=288) were excluded leaving 30,506 participants for analyses (Figure 1). The procedures followed were in accordance with the ethical standards of the responsible committee on human experimentation of China Medical University, and written informed consent of all subjects or their agents was obtained.

**Figure 2:**
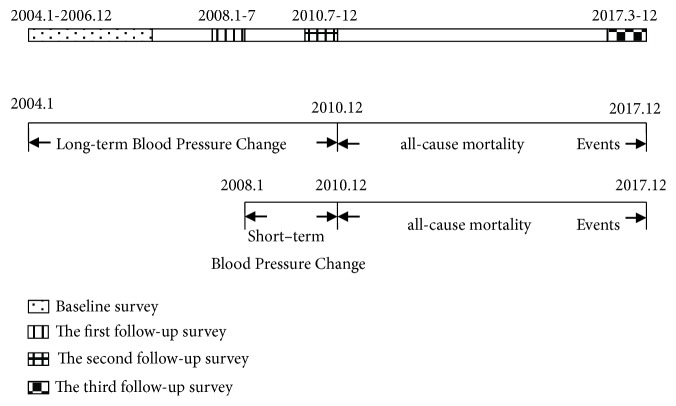
Study designs used for analysis of the associations between short- and long-term changes.

We aim to compare the association between short- and long-term BP changes and all-cause and CVD mortality (Figure 2). Long-term BP changes were between baseline and follow-up 2, and short-term BP changes were between follow-up 1 and follow-up 2. Data on events were all collected from 2010 to follow-up 2.

### 2.2. Baseline Measurement

Data on demographic variables (age, sex, and race), current drinking [14], current smoking, physical activity, history of disease (stroke, coronary heart disease (CHD), family history of hypertension, diabetes, and hyperlipemia), and information on antihypertensive medications were obtained by interview with a standard epidemiological questionnaire.

Details of the BP measurements have been described elsewhere [13]. In this study, based on the Seven Joint National Committee (JNC7) [15], we divided BP into normotension, prehypertension, and hypertension (SBP / DBP<120 / <80 mmHg, 120-129/80-89, ≥ 140 / 90 mmHg or receiving antihypertensive medications). Next, we classified the participants according to changes in BP: group 1, maintained at normotension; group 2, from normotension to prehypertension; group 3, from normotension to hypertension; group 4, from prehypertension to normotension; group 5, maintained at prehypertension; group 6, from prehypertension to hypertension; group 7, from hypertension to normotension; group 8, from hypertension to prehypertension; group 9, maintained at hypertension; the population was divided into 9 groups.

### 2.3. Follow-Up

All subjects were invited to attend the follow-up. A total of 42,242 patients finished at least one time follow-up (follow-up rate 91.5%). At each visit, we collected the information on clinical end points and concurrent medication use. During each visit, three BP measurements were taken according to a standard protocol identical to that of the baseline examination. The mean value of three BP measurements was used for each participant. We then evaluated the risk of study outcomes according to BP.

### 2.4. Study Outcomes

Our results included all-cause and CVD mortality. Deaths were confirmed through hospital records and direct contact with their families. We confirmed that death from CVD on the basis of autopsy reports, death certificates, medical record abstract, or information obtained from family members [16]. All materials were independently reviewed by the end-point assessment committee which included the certified neurologists, cardiologists, and others.

### 2.5. Statistical Analysis

Continuous variables were reported as means and standard deviations (SD), and categorical variables were expressed as frequency and percentage. The rates of events were presented as the number of events per 1000 person-years. We used multivariable Cox proportional hazards models to estimate hazard ratios (HRs) and 95% confidence intervals (CIs) for the associations between BP categories and mortality. We calculated the risk of events for participants with altered BP levels, with reference to those with unchanged BP categories. Next, we calculated the events risk of participants with changes in BP levels and made normotension BP as a reference.

We adjusted for sex, age, race, BMI, SBP, DBP, current smoking, current drinking, education level, physical activity, antihypertensive treatment, family history of hypertension, and history of diabetes, hyperlipidemia, and CVD. Beyond that, we compared the predictive power of short-term changes and long-term changes using Fisher Z test [17]. A 2-sided* P* value <0.05 was deemed significant. Moreover, receiver operating characteristic (ROC) curves were constructed, and the area under the curve (AUC) was calculated to assess the cut-off value of the SBP changes in BP elevation. All analyses were performed with SPSS statistical software version 20.0 (SPSS Inc., Chicago, Illinois, USA). A* P* value less than 0.05 was accepted as indicating statistical significance.

## 3. Results

There were 24,004 participants in short-term analysis and 30,506 participants in long-term analysis, of which 49.4% and 51.1% were women, and the mean age was 51.9 (SD, 10.8) years and 50.2 (SD, 11.0) years, respectively.  Table 1 presents the baseline characteristics of participants with short- and long-term changes in BP. For short-term, the mean (SD) of SBP and that of DBP were 131.1 (14.7) mmHg and 81.4 (10.0) mmHg, respectively. 12.7% were normotension, 54.7% were prehypertensive, and 32.6% were hypertensive. Of the long-term changes subjects, the mean BP was 133.5 (22.1)/82.3 (12.5) mmHg. The three BP categories accounted for 18.1%, 45.6%, and 36.3%, respectively.

**Figure 3:**
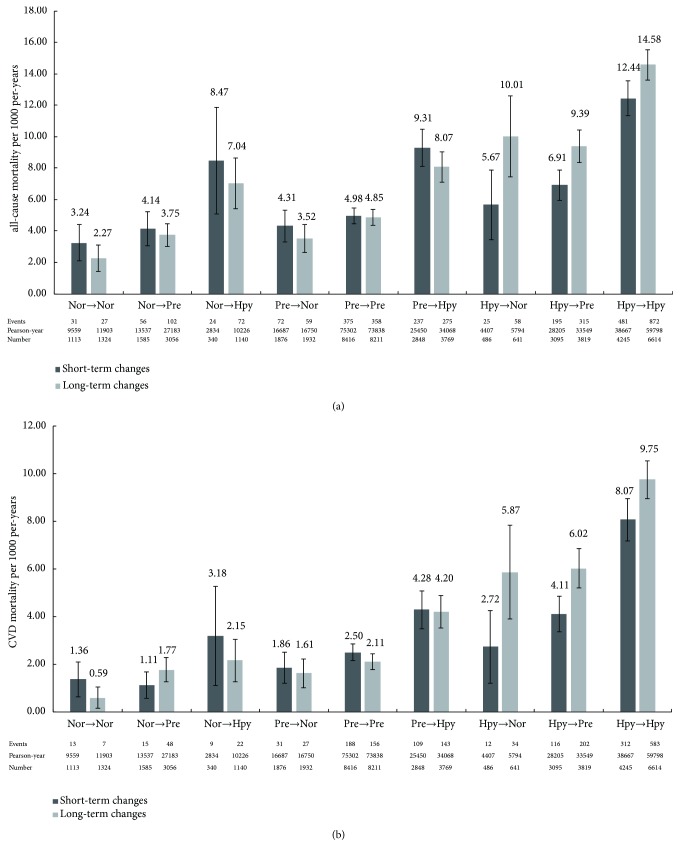
The short-term and long-term incidence of deaths and its subtypes at the different variation levels. Incident (a) all-cause mortality and (b) CVD mortality, at the different variation levels. Error bars represent 95% CI. Short-term changes: from the first follow-up (2008) to the second follow-up (2010). Long-term changes: from the baseline (2004-2006) to the second follow-up (2010). Nor: normotensive (SBP<120mmHg and DBP<80mmHg); Pre: prehypertension (120mmHg≤SBP≤139mmHg or 80≤DBP≤89mmHg); HT: hypertension (SBP/DBP≥140/90 mmHg). SBP: systolic blood pressure; DBP: diastolic blood pressure. CI: confidence interval.

Figure 3 shows the number of cases of all-cause mortality (Figure 3(a)) and CVD mortality (Figure 3(b)) per 1000 person-years by BP categories change. In the short-term BP changes study, 1496 deaths (including 805 from CVD deaths) were identified, and the overall incidence of all-cause mortality was 6.97 per 1000 person-years (CVD mortality was 3.75 per 1000 person-years). For the long-term BP changes analysis, there were 2138 all-cause mortality (1222 CVD mortality), and the total incidences of all-cause mortality and CVD mortality were 7.83/1000 person-years and 4.47/1000 person-years, respectively.

Table 2 shows HRs (95%CI) for the associations between BP changes and risk of all-cause and CVD mortality. In the multivariate adjusted Cox model for all-cause mortality, compared with BP maintainers, in short-term BP analysis, we found a significant decreasing risk of BP from hypertension to prehypertension, and the HRs (95%CI) were 0.766 (0.638-0.899) for all-cause mortality and 0.729 (0.585-0.908) for CVD mortality, respectively. And the results of women were the same; the HRs (95%CIs) were 0.606 (0.448-0.822) for all-cause mortality and 0.610 (0.418-0.889) for CVD mortality, respectively. In contrast, there were significant increasing risks of BP categories changes, and participants with BP from normotension or prehypertension to hypertension had HRs (95% CI) of 1.948 (1.118-3.392) and 1.439 (1.218-1.700) for all-cause mortality. Among men, the HRs (95% CIs) were 2.374 (1.059-5.323) and 1.672 (1.366-2.047) for all-cause mortality. The difference was a significant increasing risk of BP from prehypertension to hypertension in men, and the HR (95%CIs) was 1.401 (1.041-1.884). In long-term changes, the HRs (95% CI) of participants from normotension or prehypertension to hypertension were 1.738 (1.099-2.749) and 1.203 (1.023-1.414) for all-cause mortality, and they were 2.001 (1.041-3.838) and 1.241 (1.023-1.505) for all-cause mortality in men. BP from normotension to prehypertension had HR (95% CIs) of 2.351 (1.049-5.269) for CVD mortality. And from prehypertension to hypertension HRs (95% CIs) were 1.323 (1.047-1.672) in total and 1.354 (1.019-1.798) in men for CVD mortality. The results of nondiabetics are shown in Supplementary Appendix 1.

We also compared the effects of short- and long-term BP changes, measured as regression coefficients (*β*), and they were significantly greater in short-term changes than in long-term for all-cause mortality (total: *β*=0.667 VS *β*=0.553,* P*=0.0033; men: *β*=0.865 VS *β*=0.694,* P*<0.001) for participants from normotension to hypertension. The same result also occurs in the prehypertension to hypertension (total: *β*=0.364 VS *β*=0.185,* P*<0.001; men: *β*=0.514 VS *β*=0.216,* P*<0.001). Similarly, from hypertension to prehypertension, the results were reversed (total: *β*=-0.267 VS *β*=-0.112,* P*<0.001; women: *β*=-0.500 VS *β*=-0.104,* P*<0.001). When analyzing CVD mortality, we also found that short-term and long-term changes are different, from normotension to prehypertension (total: *β*=-0.237 VS *β*=0.855,* P*<0.001), prehypertension to hypertension (total: *β*=0.204 VS *β*=0.280,* P*=0.0012), and hypertension to prehypertension (total: *β*=-0.316 VS *β*=-0.103,* P*<0.001; women: *β*=-0.494 VS *β*=-0.126,* P*<0.001).

In Table 3, we compared all the other 8 groups with the group 1 as reference. In short-term changes, from normotension to hypertension increased significantly for all-cause mortality, the HRs (95% CI) were 1.846 (1.092-3.181). In long-term changes, there were more interesting results. For participants from normotension to hypertension the HRs (95% CIs) of all-cause mortality were 1.759 (1.129-2.742) in total and 1.892 (1.005-2.737) in men, respectively. For participants from hypertension to normotension, the HRs (95% CIs) of all-cause mortality were 1.725 (1.073-2.772). There were significant increased risks of BP categories changes normotension to prehypertension (HRs 2.409; 95%CI:1.090-5.328) and prehypertension to hypertension (HRs 2.441; 95%CI:1.136-5.244) for CVD mortality. For participants from hypertension to normotension or to prehypertension or that maintain hypertension, the HRs (95% CIs) of CVD mortality were 2.924 (1.276-6.700), 2.345 (1.083-5.081), and 2.591 (1.197-5.609), respectively. There was also a significant increased risk of BP from hypertension to normotension (HR: 3.330; 95%CIs: 1.107-10.023) for CVD mortality in women. The results of nondiabetics are shown in Supplementary Appendix 2.

**Figure 4:**
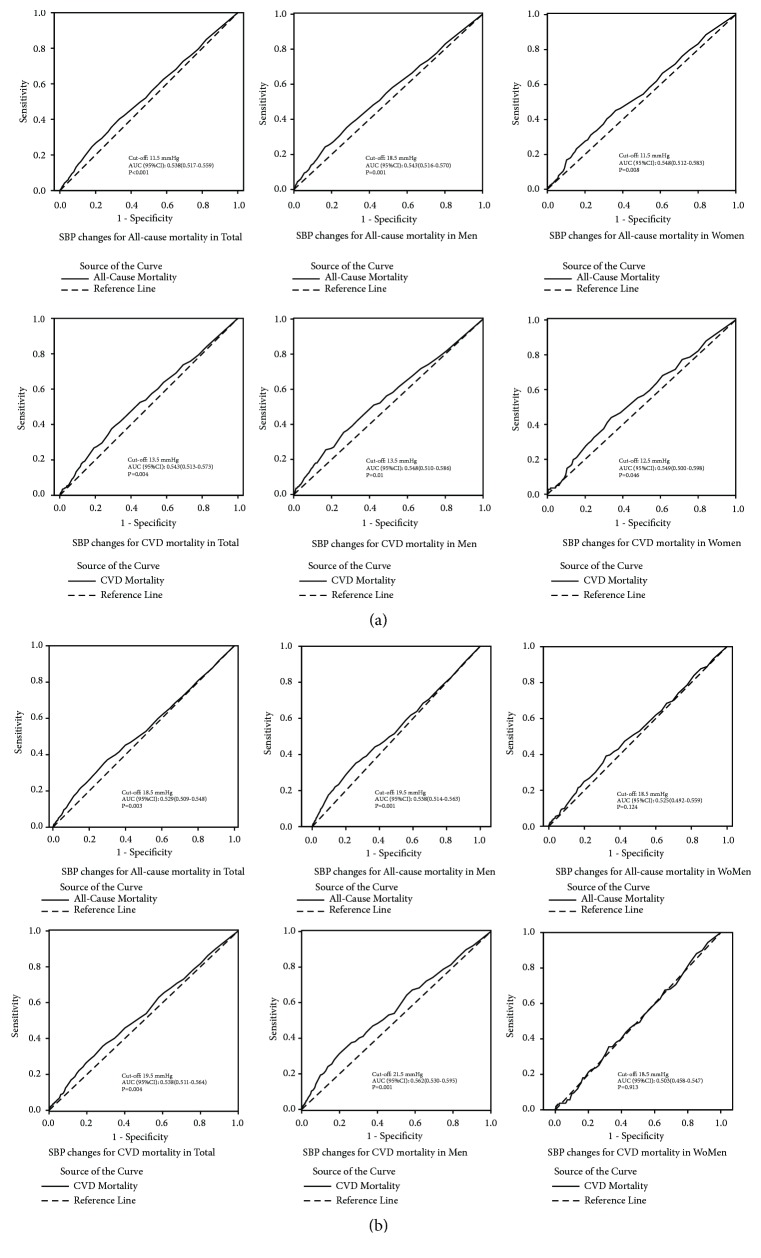
ROC curves of maximum SBP changes to predict all-cause and CVD mortality. ROC curves (a) in short-term analysis and ROC curves (b) in long-term analysis. Short-term analysis: from the first follow-up (2008) to the second follow-up (2010). Long-term analysis: from the baseline (2004-2006) to the second follow-up (2010). ROC: receiver operating characteristics; SBP: systolic blood pressure.

For participants with elevated SBP, the cut-off value of SBP changes was evaluated by using the ROC curve to predict mortality (Figure 4). In short-term analysis (Figure 4(a)), the optimal cut-off value of SBP changes for the diagnosis of all-cause mortality was 11.5 mmHg in total, and the AUC (95% CIs) was 0.538 (0.517-0.559). And the cut-off value was 13.5 mmHg for CVD mortality; the AUC (95% CIs) was 0.543 (0.513-0.573). Among men, the cut-off values of all-cause and CVD mortality were 18.5 mmHg and 13.5 mmHg, and the AUC (95% CIs) were 0.543 (0.516-0.570) and 0.548 (0.510-0.586). Among women, for all-cause mortality, the cut-off value was 11.5 mmHg, and the AUC (95% CIs) was 0.548 (0.512-0.583). For CVD mortality, the cut-off value was 12.5 mmHg, and the AUC (95% CIs) was 0.549 (0.500-0.598). In long-term analysis, the cut-off values of all-cause mortality were 18.5 mmHg (AUC: 0.529; 95% CIs: 0.509-0.548) and they were 19.5 mmHg (AUC:0.538; 95% CIs:0.511-0.564) of CVD mortality for total. Among men, the cut-off values of all-cause and CVD mortality were 19.5 mmHg (AUC:0.538; 95% CIs:0.514-0.563) and 21.5 mmHg (AUC:0.562; 95% CIs: 0.530-0.595), respectively. The results of nondiabetics are shown in Supplementary Appendix 3.

## 4. Discussion

The main findings of the present study were the positive association between short- and long-term BP changes and the risk of all-cause and CVD mortality in rural areas of China. Overall, our data showed that, compared with people who maintain the BP status, participants with elevated BP had a high risk of all-cause and CVD mortality, and participants with reduced BP had a lower risk of all-cause and CVD mortality, both in short- and long-term changes analysis. In addition, the difference between short-term and long-term changes is statistically significant.

Our study confirmed the findings for positive associations between BP changes and all-cause mortality, which was comparable with previous studies [18–21]. Two studies were performed on hypertensive patients and untreated hypertensive patients at the IPC Center in Paris, and changes in individual long-term BP are independent predictors of all-cause mortality in hypertensive patients [18]. Data from the Minnesota Business and Professional Men Study (n=261) and the Zutphen Study were shown, and the 10-year BP trajectory was the strongest predictor of cardiovascular mortality and all-cause mortality in Minnesota [19]. Cardiovascular health studies concluded that long-term visit-to-visit SBP variability was independently associated with a higher risk of subsequent mortality and a meta-analysis of 13 cohort studies in Japan also presented that adjusted mortality increased with increasing BP [20, 21]. These studies found that BP changes are a powerful predictor of cardiovascular events independently of mean SBP or DBP, which is more common in previous studies. And we also got the same conclusion when studying CVD and all-cause mortality. In addition, we explored the relationship between BP changes in the longitudinal pattern over time and the risk of subsequent mortality. What is more, we used BP short- and long-term changes to explore the relationship between BP and mortality, and this research was still very scarce in China.

We believe that short- and long-term changes in BP are an independent risk factor of mortality. And the impact of short- and long-term changes on outcomes is different. BP changes are indeed the result of a complex interaction between external environmental stimuli and the response of several cardiovascular control mechanisms [22]. There is evidence that short-term BP changes predict terminal organ damage and cardiovascular events [23–25]. However, the individual biologic mechanisms by which long-term BP changes may affect risk of mortality CVD or all-cause mortality are yet unclear. This impact might be due to changes in BP that cause significant changes in vasculature exposure to pressure load over a long period of time [26]. These would affect the potential health of vascular tissue, thereby affecting the development or severity of CVD [27, 28]. Therefore, one hypothesis is that short- and long-term changes are different for vascular pressure states. Further studies are warranted to test for this hypothesis. We detected that the same changes in BP occur, and the shorter change time seems to affect mortality more. The content of the appeal reminds us that if only one baseline BP measurement is used, the impact of hypertension on the outcome of the event will be overestimated or underestimated! Therefore, pay special attention to sudden changes in BP. Only in this way can we better help prevent cardiovascular and all-cause mortality.

Interestingly, a declining BP in hypertensive patients increases the risk of all-cause mortality, compared with maintaining normotension, especially for CVD mortality. Some studies showed that a low DBP was associated with an increased all-cause mortality risk [29, 30]. In post hoc analyses of the Systolic Hypertension in the Elderly Program (SHEP), after fully adjusting the functional status and other confounding factors, the drop in BP is still accompanied by an increase in mortality. It is also possible that lower BP in the elderly may increase the risk of adverse outcomes [31]. In assessing the prevalence of BP decline in the elderly and its relationship to subsequent outcomes, Satish S et al. [32] found that a drop in BP may be a predictor of higher mortality risk in the elderly. This result appeared more in the elderly as shown in previous studies, but the reason is unclear.

### 4.1. Strengths and Limitations

Our study had several important strengths, which include the relatively large sample size and large number of adverse events accrued, thereby increasing the statistical power of our analyses. Moreover, in this study, information about BP was derived from the mean BP of follow-up, which helped determine the relationship between BP and adverse outcomes. Finally, in addition to looking at the effect of changes in BP Categories on death, we used the ROC curve to find the cut-off value of SBP in patients with elevated BP. Our study also had several limitations. First, the BP in this study is the average of three measurements in a day, so we might not account more for the effects of individual BP fluctuations. Secondly, our research sample only included participants in rural areas of China, and we could expect different results from more people of different ethnicity. Thirdly, we did not have enough laboratory measurements, such as cholesterol, blood glucose, and inflammatory biomarkers to control these covariates. This is why we have the low frequency off diabetes in baseline characteristics. Finally, it is also worth noting that the classification of BP changes may mask some of the individual variabilities of BP in terms of time variation and may lead to attenuation.

## 5. Conclusions

In our study, using the Cox proportional hazard models with short-term BP changes and long-term BP changes entered in the same model, BP changes provided more information on risk of all-cause and cardiovascular mortality than BP at a single point in time. Our research suggests that short-term BP changes have a greater effect on mortality. And individuals who are able to maintain their BP to normal BP levels have the lowest risk for CVD and all-cause mortality. The importance of hypertension management should be widely accepted in public health practice. Prevention efforts should continue to emphasize the importance of lowering BP and maintaining normotension to reduce the mortality.

## Figures and Tables

**Table 1:** Baseline characteristics.

Characteristics	Study population 1 (n=24004)	Study Population 2 (n=30506)
Female, n (%)	11866(49.4)	15577(51.1)
Age (year)	51.9(10.8)	50.2(11.0)
Race, n (%)		
Han	18553(77.3)	23617(77.4)
Mongolian	5126(21.4)	6487(21.3)
Others	325(1.4)	402(1.3)
BMI (kg/m^2^)	23.6 (2.5)	23.2 (28)
SBP (mmHg)	131.1(14.7)	133.5(22.1)
DBP (mmHg)	81.4(10.0)	82.3 (12.5)
Education level, n (%)		
Never or Less than 5 years	9696(40.4)	13048(42.8)
Primary school	12871(53.6)	15612(51.2)
Tertiary high school or higher education	1437(6.0)	1846(6.1)
Physical activity, n (%)		
Low	6147(25.6)	8351(27.4)
Moderate	11011(45.9)	13824(45.3)
High	6846(28.5)	8331(27.3)
Current drinking, n (%)	7434(31.0)	9591(31.4)
Current smoking, n (%)	8512(35.5)	12669(41.5)
BP categories		
Normal, n (%)	3038(12.7)	5520(18.1)
Prehypertension, n (%)	13140(54.7)	13912(45.6)
Hypertension, n (%)	7826(32.6)	11074(36.3)
Family history of hypertension, n (%)	2852(11.9)	3994(13.1)
Antihypertensive treatment, n (%)	2186(9.1)	2537(8.3)
History of diabetes, n (%)	86(0.4)	137(0.4)
History of hyperlipidemia, n (%)	595(2.5)	1053(3.5)
History of CVD, n (%)	639(2.7)	1107(3.6)

Values are expressed as mean (standard deviation) or number (percentage).

BMI: body mass index; BP: blood pressure; SBP: systolic blood pressure; DBP: diastolic blood pressure; BP categories according to JNC7; study population 1: participants with short-term (2008-2010) BP category changes; Study population 2: participants with long-term (2004-2006 to 2010) BP category changes.

**Table 2:** Associations of short- and long-term BP changes with incident all-cause and CVD mortality compared with participants who maintain BP category.

BP Category at Baseline	BP Category at Follow-up	number	Short-term changes in blood pressure			number	Long-term changes in blood pressure			P values#
			Hazard Ratio (95%CI)	*P* Values	*β*		Hazard Ratio (95%CI)	*P* Values	*β*	
Total										
All-cause mortality										
Normotension	Normotension	1113	1.000 (Ref.)			1324	1.000 (Ref.)			
	Prehypertension	1585	1.256(0.793-1.990)	0.332	0.228	3056	1.379(0.895-2.125)	0.146	0.321	
	Hypertension	340	1.948(1.118-3.392)	0.019	0.667	1140	1.738(1.099-2.749)	0.018	0.553	0.0033
Prehypertension	Normotension	1876	1.057(0.818-1.366)	0.672	0.055	1932	0.930(0.704-1.228)	0.607	-0.073	
	Prehypertension	8416	1.000 (Ref.)			8211	1.000 (Ref.)			
	Hypertension	2848	1.439(1.218-1.700)	<0.001	0.364	3769	1.203(1.023-1.414)	0.025	0.185	<0.001
Hypertension	Normotension	486	0.826(0.549-1.244)	0.361	-0.191	641	1.157(0.884-1.516)	0.288	0.146	
	Prehypertension	3095	0.766(0.638-0.899)	0.002	-0.267	3819	0.894(0.782-1.021)	0.097	-0.112	<0.001
	Hypertension	4245	1.000 (Ref.)			6614	1.000 (Ref.)			
CVD mortality										
Normotension	Normotension	1113	1.000 (Ref.)			1324	1.000 (Ref.)			
	Prehypertension	1585	0.789(0.360-1.730)	0.554	-0.237	3056	2.351(1.049-5.269)	0.038	0.855	<0.001
	Hypertension	340	1.626(0.666-3.969)	0.286	0.486	1140	1.834(0.765-4.397)	0.174	0.606	
Prehypertension	Normotension	1876	0.946(0.643-1.391)	0.777	-0.056	1932	0.975(0.646-1.473)	0.906	-0.025	
	Prehypertension	8416	1.000 (Ref.)			8211	1.000 (Ref.)			
	Hypertension	2848	1.227(0.963-1.563)	0.098	0.204	3769	1.323(1.047-1.672)	0.019	0.280	0.0012
Hypertension	Normotension	486	0.639(0.355-1.147)	0.133	-0.449	641	1.105(0.778-1.568)	0.578	0.099	
	Prehypertension	3095	0.729(0.585-0.908)	0.005	-0.316	3819	0.902(0.765-1.064)	0.222	-0.103	<0.001
	Hypertension	4245	1.000 (Ref.)			6614	1.000 (Ref.)			
Men										
All-cause mortality										
Normotension	Normotension	271	1.000 (Ref.)			320	1.000 (Ref.)			
	Prehypertension	578	1.428(0.732-2.784)	0.296	0.356	1263	1.427(0.763-2.667)	0.265	0.355	
	Hypertension	117	2.374(1.059-5.323)	0.036	0.865	525	2.001(1.041-3.838)	0.037	0.694	<0.001
Prehypertension	Normotension	682	1.111(0.795-1.552)	0.538	0.105	707	1.036(0.722-1.486)	0.847	0.036	
	Prehypertension	4645	1.000 (Ref.)			4707	1.000 (Ref.)			
	Hypertension	1498	1.672(1.366-2.047)	<0.001	0.514	2182	1.241(1.023-1.505)	0.029	0.216	<0.001
Hypertension	Normotension	165	0.812(0.442-1.490)	0.501	-0.208	211	1.154(0.789-1.687)	0.460	0.143	
	Prehypertension	1723	0.853(0.692-1.052)	0.138	-0.159	1798	0.881(0.738-1.051)	0.160	-0.127	
	Hypertension	2459	1.000 (Ref.)			3216	1.000 (Ref.)			
CVD mortality										
Normotension	Normotension	271	1.000 (Ref.)			320	1.000 (Ref.)			
	Prehypertension	578	0.438(0.139-1.378)	0.158	-0.825	1263	2.927(0.888-9.643)	0.078	1.074	
	Hypertension	117	4.738(0.508-5.946)	0.379	0.553	525	3.008(0.870-10.403)	0.082	1.101	
Prehypertension	Normotension	682	1.009(0.611-1.669)	0.971	0.009	707	1.297(0.783-2.147)	0.313	0.260	
	Prehypertension	4645	1.000 (Ref.)			4707	1.000 (Ref.)			
	Hypertension	1498	1.401(1.041-1.884)	0.026	0.337	2182	1.354(1.019-1.798)	0.037	0.303	0.2585
Hypertension	Normotension	165	0.354(0.113-1.113)	0.076	-1.038	211	0.869(0.496-1.522)	0.624	-0.140	
	Prehypertension	1723	0.792(0.603-1.041)	0.095	-0.233	1798	0.907(0.726-1.131)	0.385	-0.098	
	Hypertension	2459	1.000 (Ref.)			3216	1.000 (Ref.)			
Women										
All-cause mortality										
Normotension	Normotension	842	1.000 (Ref.)			1004	1.000 (Ref.)			
	Prehypertension	1007	1.032(0.525-2.028)	0.928	0.031	1793	1.255(0.684-2.303)	0.464	0.227	
	Hypertension	223	1.515(0.675-3.401)	0.314	0.416	615	1.289(0.656-2.531)	0.461	0.254	
Prehypertension	Normotension	1194	1.033(0.691-1.545)	0.873	0.033	1225	0.851(0.549-1.321)	0.472	-0.161	
	Prehypertension	3771	1.000 (Ref.)			3504	1.000 (Ref.)			
	Hypertension	1350	1.095(0.814-1.472)	0.550	0.090	1587	1.162(0.862-1.567)	0.324	0.150	
Hypertension	Normotension	321	0.775(0.435-1.379)	0.386	-0.255	430	1.155(0.786-1.698)	0.464	0.144	
	Prehypertension	1372	0.606(0.448-0.822)	0.001	-0.500	2021	0.901(0.743-1.105)	0.318	-0.104	<0.001
	Hypertension	1786	1.000 (Ref.)			3398	1.000 (Ref.)			
CVD mortality										
Normotension	Normotension	842	1.000 (Ref.)			1004	1.000 (Ref.)			
	Prehypertension	1007	1.032(0.331-3.220)	0.956	0.032	1793	1.765(0.572-5.448)	0.323	0.568	
	Hypertension	223	1.343(0.350-5.156)	0.667	0.295	615	0.549(0.119-2.543)	0.444	-0.599	
Prehypertension	Normotension	1194	0.894(0.486-1.645)	0.719	-0.112	1225	0.697(0.340-1.426)	0.323	-0.362	
	Prehypertension	3771	1.000 (Ref.)			3504	1.000 (Ref.)			
	Hypertension	1350	0.992(0.651-1.512)	0.970	-0.008	1587	1.343(0.887-2.032)	0.163	0.295	
Hypertension	Normotension	321	0.811(0.393-1.677)	0.573	-0.209	430	1.330(0.844-2.094)	0.219	0.285	
	Prehypertension	1372	0.610(0.418-0.889)	0.010	-0.494	2021	0.882(0.686-1.133)	0.324	-0.126	<0.001
	Hypertension	1786	1.000 (Ref.)			3398	1.000 (Ref.)			

Abbreviations: normotension: subjects with blood pressure (BP) <120/80 mmHg; prehypertension: subjects with BP of 120-139/80-89 mmHg; hypertension: subjects with BP≥140/90mmHg or antihypertensive treatment. Adjusted age, gender, ethnicity, SBP, DBP, BMI, education level, physical activity, current drinking, current smoking, family history of hypertension, history of CVD diseases, history of diabetes, history of hyperlipidemia, and antihypertensive treatment.

#comparison of *β*.

**Table 3:** Associations of short- and long-term BP changes with incident all-cause and CVD mortality compared with participants relative to stable BP of normotension.

BP Category Change	Short-term changes in blood pressure			Long-term changes in blood pressure		
	Number	Hazard Ratios (95% CI)	*P* Values	Number	Hazard Ratio (95%CI)	*P* Values
Total						
All-cause mortality						
Normotension to Normotension	1113	1.000 (Ref.)		1324	1.000 (Ref.)	
Normotension to Prehypertension	1585	1.179(0.759-1.832)	0.463	3056	1.367(0.894-2.090)	0.150
Normotension to Hypertension	340	1.846(1.092-3.181)	0.023	1140	1.759(1.129-2.742)	0.012
Prehypertension to Normotension	1876	0.943(0.613-1.451)	0.790	1932	1.137(0.718-1.799)	0.592
Prehypertension to Prehypertension	8416	0.861(0.588-1.262)	0.444	8211	1.223(0.823-1.819)	0.317
Prehypertension to Hypertension	2848	1.211(0.818-1.793)	0.339	3769	1.464(0.979-2.189)	0.064
Hypertension to Normotension	486	0.726(0.415-1.272)	0.263	641	1.725(1.073-2.772)	0.022
Hypertension to Prehypertension	3095	0.672(0.437-1.032)	0.070	3819	1.323(0.873-2.007)	0.179
Hypertension to Hypertension	4245	0.893(0.581-1.372)	0.604	6614	1.488(0.982-2.255)	0.052
CVD mortality						
Normotension to Normotension	1113	1.000 (Ref.)		1324	1.000 (Ref.)	
Normotension to Prehypertension	1585	0.755(0.359-1.590)	0.460	3056	2.409(1.090-5.328)	0.030
Normotension to Hypertension	340	1.568(0.669-3.675)	0.301	1140	1.922(0.820-4.505)	0.127
Prehypertension to Normotension	1876	0.940(0.486-1.817)	0.855	1932	1.774(0.770-4.085)	0.179
Prehypertension to Prehypertension	8416	0.988(0.553-1.764)	0.966	8211	1.803(0.842-3.863)	0.126
Prehypertension to Hypertension	2848	1.200(0.661-2.177)	0.549	3769	2.441(1.136-5.244)	0.022
Hypertension to Normotension	486	0.727(0.319-1.657)	0.449	641	2.924(1.276-6.700)	0.010
Hypertension to Prehypertension	3095	0.819(0.435-1.542)	0.537	3819	2.345(1.083-5.081)	0.028
Hypertension to Hypertension	4245	1.127(0.599-2.119)	0.711	6614	2.591(1.197-5.609)	0.013
Men						
All-cause mortality						
Normotension to Normotension	271	1.000 (Ref.)		320	1.000 (Ref.)	
Normotension to Prehypertension	578	1.179(0.629-2.208)	0.607	1263	1.381(0.745-2.084)	0.306
Normotension to Hypertension	117	1.912(0.882-4.144)	0.101	525	1.892(1.005-2.737)	0.048
Prehypertension to Normotension	682	0.909(0.490-1.686)	0.762	707	1.238(0.639-1.802)	0.527
Prehypertension to Prehypertension	4645	0.775(0.445-1.349)	0.367	4707	1.214(0.677-1.807)	0.515
Prehypertension to Hypertension	1498	1.221(0.695-2.145)	0.488	2182	1.476(0.818-2.154)	0.197
Hypertension to Normotension	165	0.617(0.271-1.406)	0.250	211	1.745(0.874-2.655)	0.114
Hypertension to Prehypertension	1723	0.675(0.370-1.233)	0.201	1798	1.327(0.721-1.997)	0.363
Hypertension to Hypertension	2459	0.822(0.450-1.505)	0.526	3216	1.523(0.829-2.229)	0.175
CVD mortality						
Normotension to Normotension	271	1.000 (Ref.)		320	1.000 (Ref.)	
Normotension to Prehypertension	578	0.438(0.147-1.309)	0.139	1263	2.77(0.849-9.0360)	0.091
Normotension to Hypertension	117	1.552(0.491-4.907)	0.454	525	2.725(0.805-9.221)	0.107
Prehypertension to Normotension	682	0.735(0.303-1.782)	0.495	707	2.277(0.668-7.759)	0.188
Prehypertension to Prehypertension	4645	0.704(0.322-1.538)	0.379	4707	1.810(0.572-5.726)	0.313
Prehypertension to Hypertension	1498	0.945(0.426-2.096)	0.889	2182	2.438(0.768-7.741)	0.130
Hypertension to Normotension	165	0.267(0.067-1.071)	0.062	211	2.334(0.655-8.320)	0.191
Hypertension to Prehypertension	1723	0.612(0.265-1.415)	0.251	1798	2.388(0.743-7.676)	0.144
Hypertension to Hypertension	2459	0.779(0.337-1.802)	0.559	3216	2.634(0.821-8.455)	0.104
Women						
All-cause mortality						
Normotension to Normotension	842	1.000 (Ref.)		1004	1.000 (Ref.)	
Normotension to Prehypertension	1007	1.064(0.565-2.001)	0.848	1793	1.316(0.723-2.394)	0.368
Normotension to Hypertension	223	1.633(0.773-3.453)	0.199	615	1.514(0.789-2.908)	0.212
Prehypertension to Normotension	1194	0.922(0.500-1.701)	0.796	1225	1.008(0.528-1.924)	0.981
Prehypertension to Prehypertension	3771	0.920(0.537-1.574)	0.760	3504	1.174(0.677-2.033)	0.568
Prehypertension to Hypertension	1350	1.023(0.581-1.802)	0.937	1587	1.374(0.783-2.410)	0.268
Hypertension to Normotension	321	0.749(0.342-1.641)	0.471	430	1.604(0.829-3.103)	0.161
Hypertension to Prehypertension	1372	0.535(0.280-1.020)	0.058	2021	1.218(0.684-2.170)	0.504
Hypertension to Hypertension	1786	0.864(0.457-1.632)	0.652	3398	1.368(0.767-2.439)	0.289
CVD mortality						
Normotension to Normotension	842	1.000 (Ref.)		1004	1.000 (Ref.)	
Normotension to Prehypertension	1007	1.083(0.383-3.064)	0.880	1793	1.878(0.623-5.664)	0.263
Normotension to Hypertension	223	1.430(0.400-5.121)	0.582	615	0.693(0.155-3.102)	0.632
Prehypertension to Normotension	1194	1.157(0.429-3.116)	0.773	1225	1.240(0.380-4.048)	0.721
Prehypertension to Prehypertension	3771	1.376(0.576-3.291)	0.473	3504	1.775(0.636-4.954)	0.273
Prehypertension to Hypertension	1350	1.363(0.549-3.385)	0.504	1587	2.449(0.874-6.859)	0.088
Hypertension to Normotension	321	1.489(0.482-4.596)	0.489	430	3.330(1.107-10.023)	0.032
Hypertension to Prehypertension	1372	0.987(0.370-2.634)	0.979	2021	2.147(0.759-6.070)	0.150
Hypertension to Hypertension	1786	1.608(0.609-4.245)	0.338	3398	2.453(0.868-6.929)	0.090

Abbreviations: normotension: subjects with blood pressure (BP) <120/80 mmHg; prehypertension: subjects with BP of 120-139/80-89 mmHg; hypertension: subjects with BP≥140/90mmHg or antihypertensive treatment. Adjusted age, gender, ethnicity, SBP, DBP, BMI, education level, physical activity, current drinking, current smoking, history of CVD diseases, family history of hypertension, history of diabetes, history of hyperlipidemia, and antihypertensive treatment.

## Data Availability

The data used to support the findings of this study are available from the corresponding author upon request.

## Supplementary Materials

Supplementary Materials Supplementary Appendix 1 shows HRs (95%CI) for the associations between BP changes and risk of all-cause and CVD mortality in nondiabetics. In short-term BP analysis, we found a significant decreasing risk of BP from hypertension to prehypertension, and the HRs (95%CI) were 0.768 (0.646-0.912) for all-cause mortality and 0.729 (0.584-0.909) for CVD mortality, respectively. In contrast, there were significant increasing risks of BP categories changes, and participants with BP from normotension or prehypertension to hypertension had HRs (95% CI) of 1.948 (1.118-3.392) and 1.437 (1.216-1.699) for all-cause mortality. In long-term changes, the HRs (95% CI) of participants from normotension or prehypertension to hypertension were 1.740 (1.000-2.751) and 1.194 (1.015-1.405) for all-cause mortality. BP from normotension to prehypertension had HR (95% CIs) of 2.351 (1.049-5.269) for CVD mortality. And from prehypertension to hypertension, HRs (95% CIs) were 1.326 (1.048-1.677) for CVD mortality. The effects of short- and long-term BP changes, measured as regression coefficients (*β*), were significantly greater in short-term changes than in long-term for all-cause mortality (*β*=0.667 VS *β*=0.554, P<0.001) for participants from normotension to hypertension. The same result also occurs in the prehypertension to hypertension (*β*=0.363 VS *β*=0.178, P<0.001). Similarly, from hypertension to prehypertension, the results were reversed (*β*=-0.264 VS *β*=-0.104, P<0.001). When analyzing CVD mortality, we also found that short-term and long-term changes are different, from normotension to prehypertension (*β*=-0.237 VS *β*=0.855, P<0.001), prehypertension to hypertension (*β*=0.211 VS *β*=0.282, P=0.0024), and hypertension to prehypertension (*β*=-0.317 VS *β*=-0.093, P<0.001). In Supplementary Appendix 2, in short-term changes, from normotension to hypertension increased significantly for all-cause mortality, the HRs (95% CI) were 1.848 (1.083-3.154). In long-term changes, for participants from normotension to hypertension the HRs (95% CIs) of all-cause mortality were 1.755 (1.125-2.737). For participants from hypertension to normotension, the HRs (95% CIs) of all-cause mortality were 1.646 (1.020-2.655). There were significant increased risks of BP categories changes normotension to prehypertension (HRs 2.403; 95%CI: 1.087-5.313) and prehypertension to hypertension (HRs 2.407; 95%CI: 1.120-5.173) for CVD mortality. For participants from hypertension to normotension or to prehypertension or that maintain hypertension, the HRs (95% CIs) of CVD mortality were 2.880 (1.255-6.612), 2.339 (1.079-5.070), and 2.563 (1.183-5.551), respectively. In Supplementary Appendix 3, in short-term analysis, the optimal cut-off value of SBP changes for the diagnosis of all-cause mortality was 12.5 mmHg, and the AUC (95% CIs) was 0.537 (0.515-0.558). And the cut-off value was 13.5 mmHg for CVD mortality; the AUC (95% CIs) was 0.542 (0.512-0.572). In long-term analysis, the cut-off values of all-cause mortality were 18.5 mmHg (AUC: 0.530; 95% CIs: 0.510-0.550), and they were 19.5 mmHg (AUC: 0.542; 95% CIs: 0.516-0.569) of CVD mortality.
