# Supplementary material for: Short-Term and Long-Term Blood Pressure Changes and the Risk of All-Cause and Cardiovascular Mortality
Source: Biomed Res Int. 2019 Aug 6;2019:5274097. doi: 10.1155/2019/5274097 (PMC6699331; doi:10.1155/2019/5274097)
Supplement: Supplementary Materials — Supplementary Appendix 1 shows HRs (95%CI) for the associations between BP changes and risk of all-cause and CVD mortality in nondiabetics. In short-term BP analysis, we found a significant decreasing risk of BP from hypertension to prehypertension, and the HRs (95%CI) were 0.768 (0.646-0.912) for all-cause mortality and 0.729 (0.584-0.909) for CVD mortality, respectively. In contrast, there were significant increasing risks of BP categories changes, and participants with BP from normotension or prehypertension to hypertension had HRs (95% CI) of 1.948 (1.118-3.392) and 1.437 (1.216-1.699) for all-cause mortality. In long-term changes, the HRs (95% CI) of participants from normotension or prehypertension to hypertension were 1.740 (1.000-2.751) and 1.194 (1.015-1.405) for all-cause mortality. BP from normotension to prehypertension had HR (95% CIs) of 2.351 (1.049-5.269) for CVD mortality. And from prehypertension to hypertension, HRs (95% CIs) were 1.326 (1.048-1.677) for CVD mortality. The effects of short- and long-term BP changes, measured as regression coefficients (β), were significantly greater in short-term changes than in long-term for all-cause mortality (β=0.667 VS β=0.554, P<0.001) for participants from normotension to hypertension. The same result also occurs in the prehypertension to hypertension (β=0.363 VS β=0.178, P<0.001). Similarly, from hypertension to prehypertension, the results were reversed (β=-0.264 VS β=-0.104, P<0.001). When analyzing CVD mortality, we also found that short-term and long-term changes are different, from normotension to prehypertension (β=-0.237 VS β=0.855, P<0.001), prehypertension to hypertension (β=0.211 VS β=0.282, P=0.0024), and hypertension to prehypertension (β=-0.317 VS β=-0.093, P<0.001). In Supplementary Appendix 2, in short-term changes, from normotension to hypertension increased significantly for all-cause mortality, the HRs (95% CI) were 1.848 (1.083-3.154). In long-term changes, for participants from normoten [file 5274097.f1.doc]

Schedule 1 Associations of Short- and Long-term Bpchanges with incident all-cause and CVD mortality compare with participants who maintain BP category in Non-diabetics

| BP Category at Baseline | BP Category at Follow-up | number | Short-term changes in blood pressure | | | number | Long-term changes in blood pressure | | | *P values#* |
| --- | --- | --- | --- | --- | --- | --- | --- | --- | --- | --- |
|  |  |  | Hazard Ratio (95%CI) | *P Values* | β |  | Hazard Ratio (95%CI) | *P Values* | β |  |
| all-cause mortality | | |  |  |  |  |  |  |  |  |
| Normotension | Normotension | 1112 | 1.000 (Ref.) |  |  | 1323 | 1.000 (Ref.) |  |  |  |
|  | Prehypertension | 1581 | 1.256(0.793-1.990) | 0.332 | 0.228 | 3053 | 1.375(0.892-2.119) | 0.149 | 0.318 |  |
|  | Hypertension | 339 | 1.948(1.118-3.392) | 0.019 | 0.667 | 1132 | 1.740(1.100-2.751) | 0.018 | 0.554 | ＜0.001 |
| Prehypertension | Normotension | 1874 | 1.058(0.819-1.367) | 0.668 | 0.056 | 1927 | 0.936(0.708-1.236) | 0.640 | -0.066 |  |
|  | Prehypertension | 8399 | 1.000 (Ref.) |  |  | 8197 | 1.000 (Ref.) |  |  |  |
|  | Hypertension | 2828 | 1.437(1.216-1.699) | ＜0.001 | 0.363 | 3754 | 1.194(1.015-1.405) | 0.032 | 0.178 | ＜0.001 |
| Hypertension | Normotension | 478 | 0.770(0.499-1.188) | 0.238 | -0.261 | 636 | 1.118(0.848-1.474) | 0.430 | 0.112 |  |
|  | Prehypertension | 3085 | 0.768(0.646-0.912) | 0.003 | -0.264 | 3795 | 0.901(0.789-1.030) | 0.126 | -0.104 | ＜0.001 |
|  | Hypertension | 4222 | 1.000 (Ref.) |  |  | 6552 | 1.000 (Ref.) |  |  |  |
| CVD mortality | | |  |  |  |  |  |  |  |  |
| Normotension | Normotension | 1112 | 1.000 (Ref.) |  |  | 1323 | 1.000 (Ref.) |  |  |  |
|  | Prehypertension | 1581 | 0.789(0.360-1.730) | 0.554 | -0.237 | 3053 | 2.351(1.049-5.269) | 0.038 | 0.855 | ＜0.001 |
|  | Hypertension | 339 | 1.626(0.666-3.969) | 0.286 | 0.486 | 1132 | 1.834(0.765-4.397) | 0.174 | 0.606 |  |
| Prehypertension | Normotension | 1874 | 0.948(0.644-1.394) | 0.786 | -0.054 | 1927 | 0.980(0.649-1.480) | 0.923 | -0.020 |  |
|  | Prehypertension | 8399 | 1.000 (Ref.) |  |  | 8197 | 1.000 (Ref.) |  |  |  |
|  | Hypertension | 2828 | 1.235(0.969-1.574) | 0.088 | 0.211 | 3754 | 1.326(1.048-1.677) | 0.019 | 0.282 | 0.0024 |
| Hypertension | Normotension | 478 | 0.625(0.340-1.150) | 0.131 | -0.469 | 636 | 1.101(0.771-1.571) | 0.598 | 0.096 |  |
|  | Prehypertension | 3085 | 0.729(0.584-0.909) | 0.005 | -0.317 | 3795 | 0.911(0.772-1.075) | 0.269 | -0.093 | ＜0.001 |
|  | Hypertension | 4222 | 1.000 (Ref.) |  |  | 6552 | 1.000 (Ref.) |  |  |  |

Abbreviations: Normotension: subjects with blood pressure (BP) ＜120/80 mmHg; Prehypertension: subjects with BP of 120-139/80-89 mmHg; Hypertension: subjects with BP≥140/90mmHg or antihypertensive treatment. adjusted age, gender, ethnicity, SBP, DBP, BMI, education level, physical activity, current drinking, current smoking, family history of hypertension, history of CVD diseases, history of hyperlipidemia, antihypertensive treatment.

#comparison of β
